# Mxene-bpV plays a neuroprotective role in cerebral ischemia-reperfusion injury by activating the Akt and promoting the M2 microglial polarization signaling pathways

**DOI:** 10.1007/s10856-024-06811-0

**Published:** 2024-07-29

**Authors:** Jing Cheng, Han Yu, Zhi-Feng Zhang, Hong-Xiang Jiang, Ping Wu, Zhou-Guang Wang, Zhi-Biao Chen, Li-Quan Wu

**Affiliations:** 1 Department of Neurosurgery, Renmin Hospital of Wuhan University, 99 Zhang Zhidong Street, Wuhan, 430060 China; 2 Department of Pathology, Xiangyang No.1 People’s Hospital, Key Laboratory of Zebrafish Modeling and Drug Screening for Human Diseases of Xiangyang City, Department of Obstetrics and Gynaecology, Hubei Provincial Clinical Research Center for Accurate Fetus Malformation Diagnosis, Hubei University of Medicine, Xiangyang, 441000 China; 3 Department of Physiology, School of Basic Medical Sciences, Hubei University of Medicine, Shiyan, 442000 China; 4 Oujiang Laboratory (Zhejiang Lab for Regenerative Medicine, Vision, and Brain Health), School of Pharmaceutical Sciences, Wenzhou Medical University, Wenzhou, 325000 China

**Keywords:** Mxene(Ti_3_C_2_T_x_), bpV (HOpic), PTEN, ischemia-reperfusion injury, M2 microglial polarization

## Abstract

Studies have shown that the inhibition of phosphatase and tensin homolog deleted on chromosome 10 (PTEN)was neuroprotective against ischemia/reperfusion(I/R) injury. Bisperoxovanadium (bpV), a derivative of vanadate, is a well-established inhibitor of PTEN. However, its function islimited due to its general inadequacy in penetrating cell membranes. Mxene(Ti_3_C_2_T_x_) is a novel two-dimensional lamellar nanomaterial with an excellent ability to penetrate the cell membrane. Yet, the effects of this nanomaterial on nervous system diseases have yet to be scrutinized. Here, Mxene(Ti_3_C_2_T_x_) was used for the first time to carry bpV(HOpic), creating a new nanocomposite Mxene-bpV that was probed in a cerebral I/R injury model. The findings showed that this synthetic Mxene-bpV was adequately stable and can cross the cell membraneeasily. We observed that Mxene-bpV treatment significantly increased the survival rate of oxygen glucose deprivation/reperfusion(OGD/R)--insulted neurons, reduced infarct sizes and promoted the recovery of brain function after mice cerebral I/R injury. Crucially, Mxene-bpV treatment was more therapeutically efficient than bpV(HOpic) treatment alone over the same period. Mechanistically, Mxene-bpV inhibited the enzyme activity of PTEN in vitro and in vivo. It also promoted the expression of phospho-Akt (Ser^473^) by repressing PTEN and then activated the Akt pathway to boost cell survival. Additionally, in PTEN transgenic mice, Mxene-bpV suppressed I/R-induced inflammatory response by promoting M2 microglial polarization through PTEN inhibition. Collectively, the nanosynthetic Mxene-bpV inhibited PTEN’ enzymatic activity by activating Akt pathway and promoting M2 microglial polarization, and finally exerted neuroprotection against cerebral I/R injury.

**Graphical Abstract:**

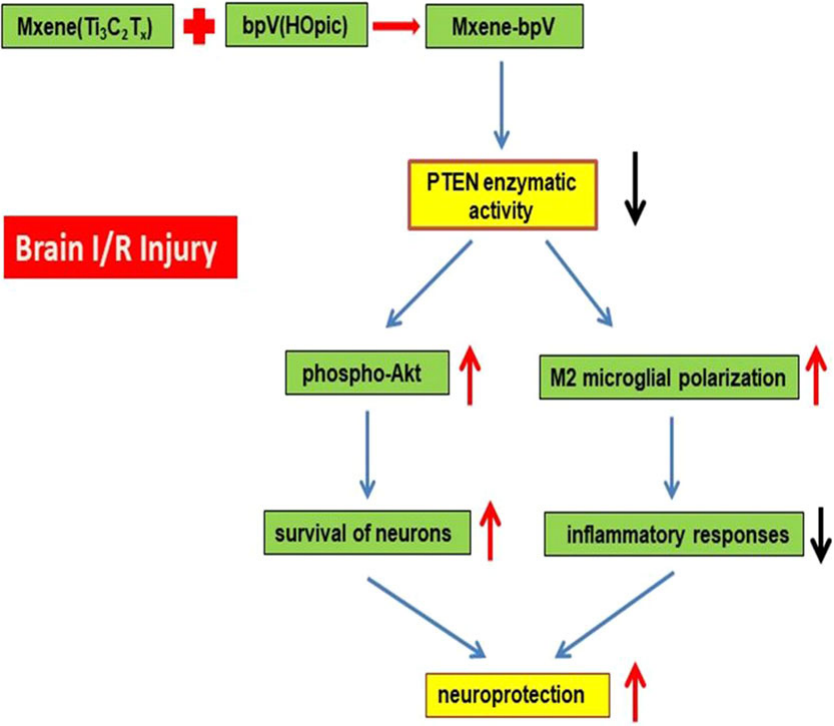

An ischemic stroke is a cerebrovascular disease with high disability and mortality outcomes. It is a severe brain injury disease caused by insufficient cerebral blood flow supply stemming from cerebral vascular obstruction. Ischemic strokes account for 60–70% of all stroke [1, 2], and their pathogenesis is a result of cerebral embolism triggered by insufficient cerebral blood flow supply, resulting in cerebral hypoxia and glucose deficiency and eventually to brain damage. Timely blood reperfusion is a key approach to reducing ischemic stroke-related damages. However, restoring blood supply could lead to ischemia/reperfusion(I/R) injury in ischemic brain tissues, which would aggravate irreversible brain damage further [3–5]. Significant neuronal damages and deaths are key factors in the aggravation of brain tissue injuries after ischemia reperfusion. Therefore, exploring the pathogenesis of cerebral ischemia-reperfusion injuries, as well as effective therapeutic targets for the treatment of ischemic stroke and improving the prognoses of patients, is of great significance.

The rapid growth in multidisciplinary nanomedicine and nanobiology has seen multiple nanocarriers developed for the diagnosis and treatment of diseases. In recent years, two-dimensional (2D) materials have been studied extensively, starting with the identification of the unique physical properties of single-layer graphene. The interest in this subject has led to a new wave of research on known 2D materials and the discovery of many new 2D materials, such as Mxene. Mxene is a novel two-dimensional lamellar nanomaterial obtained by etching its precursor MAX phase using hydrogen fluoride. While previous assessments of Mxene have established its excellent biocompatibility, making Mxene a potential universal drug carrier for the treatment of nervous system diseases, no inquiry has yet focused on its therapeutic effects on neurological disorders.

Vanadium Bisperoxovanadium (bpV), a derivative of vanadate, is a recognized inhibitor of phosphatase and tensin homolog deleted on chromosome 10 (PTEN) [6]. Considerable evidence suggests that inhibiting PTEN is neuroprotective against ischemia/reperfusion injury [7–10]. In this study, for the first time, Mxene (Ti_3_C_2_T_x_) was loaded with bpV (HOpic)(Chemical Name:(5-hydroxy-2-pyridinecarboxylato-κN1,κO2)oxodiperoxy-vanadate(2-), dipotassium), creating a new nanocomposite Mxene (Ti_3_C_2_T_x_)- (5-hydroxy-2-pyridinecarboxylato-κN1,κO2)oxodiperoxy-vanadate(2-), dipotassium(Mxene-bpV) for the treatment of cerebral I/R injury. Treatment with the nanocomposite improved the survival rate of mice with oxygen glucose deprivation/reperfusion(OGD/R)-injured neurons and reduced the size of cerebral infarction in mice. More importantly, Mxene-bpV treatment achieved better therapeutic outcomes than bpV(HOpic) treatment alone over the same period.

Here, the neuroprotective mechanism of Mxene-bpV, which comprises inhibiting PTEN activity, activating the Akt signaling pathway to promote cell survival, and promoting M2 microglial polarization to prevent brain inflammation after I/R injury, is disclosed. The results provide key experimental and theoretical bases for exploring a new strategy for the treatment of ischemic strokes.

## Materials and methods

### Materials

bpV (HOpic) and Mxene (Ti_3_C_2_T_x_) were obtained from MedChemExpress Co. Ltd (Shanghai, China) and Qiyuebio Co. Ltd (Xi’anChina), respectively.

### Synthesis and characterization of Mxene-bpV

First, Mxene-bpV drug-loaded nanoparticles were formed via electrostatic adsorption between Mxene and bpV. FTIR spectra were recorded with a VERTEX 70 spectrometer (Bruker, Germany) at room temperature, and high-resolution transmission electron microscopy (HR-TEM) was achieved using a Talos F200X (FEI, The Netherlands)microscope operating at a voltage of 200 kV. The element distribution of nanosheets was characterized using the Energy spectrometer (EDS), and the crystalline phases of nanosheets and compounds were evaluated employing an X-ray diffractometer (XRD) (SmartLab-SE, Japan).

### Animals

Male C57BL/6 J mice (8-10 weeks old, the weight fluctuated around 25 g + 1.5 g) were housed in a temperature-controlled room (23–25 °C) with a 12 h light/dark cycle and free access to drinking water and food for adaption to the environment for at least 7 days before the experiment. All animal use and experimental protocols were approved and carried out in compliance with the Institutional Animal Care and Use Committee (IACUC) guidelines and the Animal Care and Ethics Committee of Wuhan University School of Medicine. Usinga random method, samples were assigned, and data were collected and processed. All animal-related experiments were conducted following the Animal Research: Reporting of In Vivo Experiments(ARRIVE) guidelines [11].

### Intraventricular injection (i.c.v.)

Lateral ventricles in mice were located anatomically (2 mm posteriorly to the anterior fontanel, 1.5 mm lateral to the sagittal suture, and 2.5 mm downward to the skull surface) first, using a 23-gauge needle connected to a Hamilton microsyringe through a polyethylene tube. Needle placement in the ventricle was confirmed upon the removal of several microliters of clear cerebrospinal fluid using a microsyringe. Specific concentrations of the Mxene-bpV mixture or bpV(HOpic) prepared with normal saline was injected into the lateral ventricles of the mice at a rate of 1.0 μl/minafter middle cerebral artery occlusion 90 min/reperfusion.

### Local cerebral ischemia

Transient cerebral ischemia was induced in this research using the thread embolism technique [12]. Male C57BL/6 J mice in each group were selected randomly and, amongst these, those with the same body weight range were picked for the experiment. The mice were treated with 4% isoflurane in 30%O_2_ and 70% N_2_O, after which an incision was made on the median neck, and the right external carotid artery (ECA) was exposed and dissected carefully before the occlusion of the right internal carotid artery (MCA) (approximately 22 mm) from the ECA insertion line to the right internal carotid artery. 90 min after occlusion, the embolus thread was removed to allow reperfusion. The ECA was then ligated, and the wound was closed by suturing. Body temperature was kept at 37.0 ± 0.5 °C using a heating pad and a heat lamp.

24 h after MCAO/R, the mice were anesthetized with 4% isoflurane and the administration of 70% N_2_O and 30% O_2_ through inhalation, followed by reperfusion with ice-cold 0.9% saline. The brains were subsequently removed immediately and stored in the refrigerator.

### 2,3,5-triphenyltetrazolium chloride(TTC) staining

Mice were sacrificed, and their brain tissues were harvested, TTC-stained, an examined for cerebral infarct volumes. The brains, once removed, were placed in a cooled substrate and cut into 2-mm coronal sections. Each section was placed in a 10-cm dish and incubated with a mixture of phosphate buffer and 2% TTC in an oven at 37 °C for 30 min. After the incubation, the sections were fixed in 4% paraformaldehyde and stored in a refrigerator overnight at 4 °C. The ImageJ software was used to scan images of brain tissue sections and analyze them. All images were acquired, processed, and analyzed blindly. Cerebral infarct size ratio = cerebral infarct size/total area of brain slices in each group [13].

### Primary mice cortical neuron culture and OGD/R insult

Cortical neuronal cultures were prepared from 17 days pregnant C57BL/6 J mice, as described previously [14]. Pregnant mice were anesthetized using 4% isoflurane, 30% O_2_ and 70% N_2_O and then sacrificed by cervical dislocation. After whole-body sterilization of the mice with 70% ethanol, the embryos were removed and placed in a meningeal plating medium (neural basal medium, 2% B-27 supplement, 0.5% FBS, 0.5 mM L-glutamax and 25 mM glutamate), where the embryonic brains were broken rapidly, and embryonic cortical brain tissues were harvested and kept at low temperature. Mice cortical neurons were seeded in poly-D-lysine-coated dishes and suspended in a culture medium. Every 3 days thereafter, half the medium in the plate was removed in the same manner and replaced with a maintenance medium (Neurobasal medium, 2% B-27 supplement, and 0.5 mM L-glutamine). Neurons cultured for 12 days were later used for further experiments.

For the OGD/R injury, the nerve cells were transferred into adeoxygenated glucose-free extracellular solution containing 116 mmol/L NaCl, 0.8 mmol/L MgSO_4_, 1.8 mmol/L CaCl_2_, 1.0 mmol/L NaH_2_PO_4_, 5.4 mmol/L KCl and 26 mmol/L NaHCO_3_ and cultured in a specialized humidification chamber maintained in 85% N_2_, 5% CO_2_ and 10% H_2_ at 37 °C for 60 min. During the recovery process, a fresh maintenance medium containing appropriate concentrations of reagents was introduced to replace the old medium, and the mixture was left to culture in a 5% CO_2_ and 95% O_2_ incubator for 24 h.

### Cortical microglia culture

17 days pregnant C57BL/6 J mice were selected for the culture of mice cortical microglia. After whole-body sterilization of the mice with 70% ethanol, embryos were harvested. The mice were anesthetized using 4% isoflurane, 30% O_2_ and 70% N_2_O and then sacrificed by cervical dislocation. The embryos were then taken out of the uteri of pregnant mice, and their brain tissues were swiftly isolated. Cell pellets were cultured and incubated in a DMEM medium containing 5 ng/ml vector-free recombinant mouse GM-CS, 1% antibiotic-antifungal drugs and 10% fetal bovine serum at 37 °C. About 1.3*10^6^ cells were then added to a poly-D-lysine-coated T75 cell culture treatment flask and placed in an incubator at 37 °C. The culture supernatant was replaced with 10 ml of a fresh medium every 3 days, and, after about 3 weeks, the purity of microglia was registered at more than 90%, and the next experiment was performed.

### Western blotting

Western blotting was performed as described previously [15]. The polyvinylidenedifluoride membrane (Millipore, USA) was incubated with primary antibodies overnight at 4 °C. (The primary antibodies used in this experiment included: PTEN mouse monoclonal antibody, 1:1000, cat. no. sc-7974; Santa Cruz Biotechnology; Akt mouse monoclonal antibody, 1:1000, cat. no. sc-5298; Santa Cruz Biotechnology; phospho-Akt (Ser^473^) mouse monoclonal antibody, 1:1000, cat. no. sc-293125; Santa Cruz Biotechnology; GAPDH mouse monoclonal antibody, 1: 3000, cat. no. sc365062; Santa Cruz Biotechnology.). The membranes were next labeled with horseradish peroxidase-conjugated secondary antibodies (goat anti-mouse IgG 1:10,000; cat. no. ab205719; Abcam), and protein bands were imaged using a SuperSignal West Femto Maximum Sensitivity Substrate (Pierce, USA). Blot images were obtained directly from polyvinylidenedifluoride membranes using an EC3 imaging system (UVP, USA). To the experimenter, the group assignments during the experiment were blinded. The quantitative analysis of the Western blotting-obtained data was performed using the ImageJ software.

### Immunohistochemistry

Mice were overdosed with isoflurane, and their hearts were perfused – first, with 0.9% saline, then in 4% paraformaldehyde (PFA) for 24 h at 4 °C, and lastly in 30% sucrose in 0.1 mol/L phosphate buffer for 72 h at 4 °C. After all that, the brain tissues of the mice were harvested, stored in a 4% paraformaldehyde solution overnight at 4 °C, and cut into 16-μm coronal sections using a Leica VT1000S vibrating knife (Leica Micro-systems AG, Nussloch, Germany). The brain sections were incubated with primary antibodies, mouse anti-PTEN (1:100)(Santa Cruz Biotech, USA) and rabbit anti-NeuN (neuron-specific nuclear protein)(Abcam, USA), and secondary antibodies (goat anti-rabbit 488 and goat anti-mouse 594) obtained from Eugene Molecular Probes, USA. Sections were captured by investigators blinded to the experiment using an Olympus fluorescence microscope (IX51, Olympus, Japan). The Image J software (Image J, USA) was used for analysis.

### PTEN activity assay

This assay was conducted as described previously [16]. The ability of Mxene-bpV to inhibit PTEN activity was assessed using the malachite green phosphatase assay kit (Echelon Biosciences, Salt Lake City, UT, USA) following the manufacturer’s instructions. The PTEN enzyme and PIP_3_ substrate were purchased from Echelon Biosciences (Salt Lake City, UT). According to the principle of the detection method used here, PI(3,4,5)P_3_ can form a phosphate colored complex with molybdate/malachite green after 20 minat room temperature. This complex is then analyzed quantitatively, and the absorbance is read at 620 nm.

Control Wells containing PIP_3_ were subsequently set to 0% PTEN activity, and the Wells containing PTEN + PIP_3_ were set to 100% PTEN activity. The percentage conversion of PIP_3_ was determined using the formula provided in the manufacturer’s instructions, and the concentration response curve was drawn utilizing the GraphPad Prism 5 software to calculate IC_50_ values.

### Mxene-bpV membrane permeability

This assay was performed as described previously [16]. SH-SY5Y cells were deposited in 60-mm dishes at 8 × 10^5^ cells/dish, and, upon reaching 80-90% fusion, they were subjected to an extracellular solution, washed three times and then treated with Mxene-bpV for 30 min. The extracellular fluid consisted of 5.4 mM KCl, 137 mM NaCl, 1.0 mM MgCl_2_, 2.0 mM Ca^2+^, 33 mM glucose and 25 mM HEPES titrated to pH 7.4 with an osmolarity of 300 to 320 mOsm. Next, the excess Mxene-bpV was washed away with an extracellular solution, and the cells were incubated with 0.25% trypsin for 1 min, resuspended in 2 ml DMEM, centrifuged at 1000 × *g* for 3 min, and the supernatant was removed, and the precipitate resuspended in 200 μl of 0.9% saline for sonication. The sonicated mixture was then centrifuged at 14,000 × *g* for 20 min at 4 °C, and the precipitate was separated. Lastly, a double volume of methanol was added to the retained supernatant and centrifuged for deproteinization.

The synthesized [^13^C]-Mxene-bpV(Jiangyin Beta Medical Technology Co., LTD., China) was dissolved in a mixture of water and acetonitrile (1:1 volume ratio of water to acetonitrile) to a concentration of 5 μM. 20 μl of the previously collected supernatant was mixed with 20 μl of the 5 μM [^13^C]-Mxene-bpV solution, and the mixture was transferred into a 500 μl centrifuge tube. 5 μl of that mixture was injected into the Liquid Chromatograph-Mass Spectrometer (LC-MS) system, and the signal intensities of [^13^C]-Mxene-bpV and Mxene-bpV in the mixture were measured. The intensity ratio of the signal produced by Mxene-bpV to that produced by [^13^C]-Mxene-bpV (internal standard) was calculated. The ratio of the Mxene-bpV signal intensity to the [^13^C]-Mxene-bpV signal intensity was obtained by measuring the signal intensities of the Mxene-bpV and [^13^C]-Mxene-bpV mixtures. The relative intensity of [^13^C]-Mxene-bpV was 100%. A higher ratio points to more Mxene-bpV crossing the cell membrane.

### Quantitative reverse transcription-PCR

The RNA extracted from the mice and cells for use in this portion of the experiment was amplified using the TRIzol reagent (Invitrogen, USA) and then reverse transcribed with the Maxima H Minus First Strand cDNA Synthesis Kit (Thermo, USA). qRT-PCR reactions were performed utilizing the SYBR Green PCR Master Mix (Invitrogen, USA) according to the manufacturer’s instructions. Preliminary arrangements are portrayed in Table 1. The specific steps are as follows: first, 20 mg of brain tissue were introduced into the pre-cooled homogenization tube; then 1 ml of the RNA extraction solution was added to the tube, and the mixture was homogenized until no tissue mass was visible. (Alternatively, the old PBS solution in the culture bottle can be replaced with a pre-cooled PBS solution, after which the new mixture is gently shaken and washed, and the PBS solution is discarded, 1 ml of the RNA extraction solution is added, and the broken cells are blown away with a pipette). The samples were then pretreated at 12000 rpm and centrifuged for 10 min. Next, 250 µl of trichloromethane was added to the supernatant, and, after mixing, the mixture was left to react for 3 min before another centrifugation at 4 °C. The centrifugation produced 400 µl of supernatant. RNA Precipitation: 320 µl of isopropyl alcohol was added, and the mixture was blended evenly and left to react in the refrigerator at –20 °C for 15 min. The same centrifugation steps used above were applied here. The white sediment at the bottom of the centrifugation tube was the extracted RNA. RNA Washing: After the supernatant was discarded, 1.5 ml of 75% alcohol was added, and centrifugation was conducted, as provided above. Once more, the resulting supernatant was discarded. RNA dissolution: The centrifugation tube was set on a sterile work bench and blow dried. Then 15 μl of the RNA solution was added to the tube, and the blend was left to react at 55 °C for 5 min. RNA concentration determination: Nanodrop 2000 determination of the content and purity of RNA: after correcting the instrument to the zero mark, 2.5 μl of the RNA extract were introduced into the detection hole, and the absorbance of RNA was determined using a software. RNA reverse transcription to obtain cDNA: The Maxima H Minus First Strand cDNA Synthesis Kit (Thermo, USA) was used to construct a reverse transcription system alongside a 4 μl 5 x Reaction Buffer in an RNA-free PCR tube. The upper and lower primers were 0.5 μl, and 1 µl of RT enzyme mixture, respectively. The total RNA extracted (above) and RNase free water were supplemented to 20 μl, gently mixed and centrifuged. The reaction temperatures were 25 °C, 42 °C, and 85 °C, and the reaction periods were 5 min, 30 min, and 5 s, respectively. Fluorescent quantitative PCR: 0.1 ml of the RNA was introduced to each hole of a PCR reaction plate –3 holes for each sample. The constitution of the reaction system was as follows: 7.5 μl 2×SYBR Green qPCR Master Mix (None ROX), 1.5 μl F/R Primers, 2.0 μl cDNA, 4.0 μl Water Nuclease-Free. Reaction conditions: Pre-denaturation at 95 °C for 30 seconds, then denaturation at 95 °C for 15 seconds and annealing/extension at 65 °C for 30 seconds for a total of 40 cycles. At the end, fluorescence signals were determined with every 0.5 °C rise in temperature (from 65 °C to 95 °C). Quantitative analysis was carried out using the 2-△△CT method. Cycle time values were normalized to GAPDH levels in the same assay. Each assay was independently repeated thrice.

**Table 1:** Primers for quantitative reverse transcription-PCR polymerase chain reaction

Gene	Primer sequence(5′-3′)
GAPDH	Forward:5′-GCACCGTCAAGGCTGAGAAC-3′ Reverse:5′-TGGTGAAGACGCCAGTGGA-3′
CD86	Forward:5′- ACAGCAAAAGACACCCACGG-3′ Reverse:5′- CTTGTTTCATTCTGAGCCTCCTC-3′
CD32	Forward:5′-AATCCTGCCGTTCCTACTGATC-3′ Reverse:5′9-GTGTCACCGTGTCTTCCTTGAG-3′
Arg-1	Forward:5′-TCACCTGAGCTTTGATGTCG-3′ Reverse:5′-CTGAAAGGAGCCCTGTCTTG-3′
CD206	Forward:5′-CAAGGAAGGTTGGCATTTGT-3′ Reverse:5′-CCTTTCAGTCCTTTGCAAGC-3′
YM-1	Forward:5′-CAGGGTAATGAGTGGGTTGG-3′ Reverse:5′-CACGGCACCTCCTAAATTGT-3′

### PTEN transgenic mice

Because knocking out the germline encoding PTEN is embryonic lethal, tamoxifen-induced PTEN conditional knockout was used in the mice for this inquiry. The rationale for this approach is based on tamoxifen-induced Cre recombinase and a mutated estrogen receptor ligand-binding space (CreER) to eliminate the PTEN allele on the LoxP side (PTEN^LoxP/LoxP^) under the control of the ubiquitin-expressing gene encoding the ubiquitin C (Ubc) promoter.

The treatment of PTEN^LoxP/LoxP^Ubc-CreER^+^ mice with tamoxifen resulted in the excision of the flanking exons of LoxP and then PTEN gene knockdown. The procedure was carried out as follows: first, Ubc-CreER transgenic mice from the Shanghai Model Biology Research Center department were crossed with PTEN^LoxP/LoxP^ mice from Wuhan University in the laboratory; next, genomic DNA was extracted from the tail tissues of F1 and F2 generation mice and used to genotype the CreER gene and PTEN gene for each mouse; finally, PTEN^LoxP^ and Cre were used as primers for PCR typing. PTEN^LoxP^ forward: CTCCTCTACTCCATTCTTCCC and reverse: ACTCCCACCAATGAACAAAC; Cre forward: GCGGTCTGGCAGTAAAAACTATC and reverse:GTGAAACAGCATTGCTGTCACTT. At 4–6 weeks of age, pre-mutant mice (PTEN^LoxP/LoxP^Ubc-CreER^+^) and control mice (PTEN^LoxP/LoxP^UbcCreER^−^) were perfused intraperitoneally with 1 mg of tamoxifen. Three days after the last infusion of tamoxifen, the phenotype of PTEN-deficient mice was examined. The deployed animal experimental protocol was approved by the Animal Experiment Ethics Committee of Wuhan University and implemented in accordance with the “Laboratory Animal Management Regulations of Hubei Province”.

### Enzyme linked ImmunoSorbent assay (ELISA)

ELISA kits were bought from R&D Systems, Minneapolis, Minnesota, USA. Tumor necrosis factor-α (TNF-α), interleukin-1β (IL-1β), and interleukin-6 (IL-6) levels were assessed in strict accordance with the experimental instructions provided in the manufacturer’s protocol. The specific steps were as follows: Coating. The coated diluent was added to the antibody to dilute the protein concentration to 5 µl/ml. 100 µl of the resulting mixture was then deposited in each well of the ELISA plate and placed in the refrigerator at 4 °C overnight. Next, the coated solution was introduced, and 300 µl of the PBST solution was added to each well and left to react. The reaction time was 5 min*3 times. Closure. 200 µl of 10% calf serum was added to each well, and the blend was left to incubate at room temperature for 90 min. Washing. Each well was thrice rinsed with 300 µl of the PBST liquid. Each rinse lasted 5 min. Sample addition. 100 µl of the sample to be tested was diluted with sealing solution, and introduced into each reaction well. The mixture was then left to incubate at room temperature for 2 h. Post-washing antibody addition. 100 μl of the HRP-labeled antibody diluted in an ELISA kit, was added to each reaction well and left for incubation at room temperature for 1 h. After the incubation, the mixture was washed and doused with a color developing substrate, after which 100 μl of the newly configured TMB substrate solution was introduced into the reaction air and left to react at room temperature. Obvious color gradient appears in the standard sample holes. Stopping the reaction. 100 μl of sulfuric acid was added to each reaction well to stop the reaction. Results. The OD value of each pore sample was quickly determined on the enzyme marker at 450 nm, a standard curve was sketched, and the concentration of the sample was calculated.

### Lactate dehydrogenase release and cell viability analyses

Lactate dehydrogenase (LDH) release was probed using the colorimetric CytoTox 96 cytotoxicity kit (Promega). Cell viability assays were carried out in neuronal cultures based on the ability of the neurons to take up mingestthiazolyltetrazolium (MTT) (PowerWave X; BioTek, Winooski, VT). Both tests were performed per the instructions in the manufacturer’s protocol.

### Neurobehavioral tests

#### The modified Neurological Severity score (mNSS)

The mNSS of mice were determined as described previously [17]. These scores were for battery of motor, sensory, reflex, and balance tests similar to contralateral neglect tests in humans, and they were used to grade neurological functions: from 0 to 18 (normal score, 0; Maximum defect score, 18).

#### The beam walking test

The Beam walking test is used to measure the complex neuromotor function of animals [18]. Animals were timed as they walked on a (100 × 2 cm) beam. A box was placed at one end of the beam to provide the animals with a sense of safety, and a loud noise was generated to prompt the animal to move towards and into the box. The animals were scored based on the time they took to enter the box – the higher the score, the more severe the neurological deficit.

#### The adhesive removal test

The modified adhesive tape (MST) test was used to evaluate forelimb function [19]. Using a 3.0 × 1.0 cm yellow paper tape, a sleeve was made and used to wrap the forepaws of mice so that the tape was attached to the forepaws and the fingers prostrated slightly from the sleeve. The typical response of a mouse in such a situation is to pull the tape with the mouth or brush it with the contralateral paw in a forceful attempt to remove the cannula. The wrapped mice were placed in cages and observed for 30 s. The first timer was set to run without interruption, and the second timer was initiated when the animal tried to remove the tape-made sleeve. The left (affected)/right (healthy) forelimb function ratio was recorded, and the test was repeated three times a day, with the two best scores averaged. A lower ratio indicates a more severe neurological deficit.

### Statistical analysis

The data and statistical analyses of this study were performed in accordance with the recommendations for experimental design and analysis [20]. The significance criterion (alpha) was set at 0.05. Bonferroni tests were used for appropriate post hoc comparisons, while ANOVA was used to evaluatethe statistical significance of data differences between groups, where applicable. Significance was set at *p* < 0.05. All results are presented as the mean ± SEM.

## Results

### Mxene-bpV synthesis and characterization

**Fig. 1:**
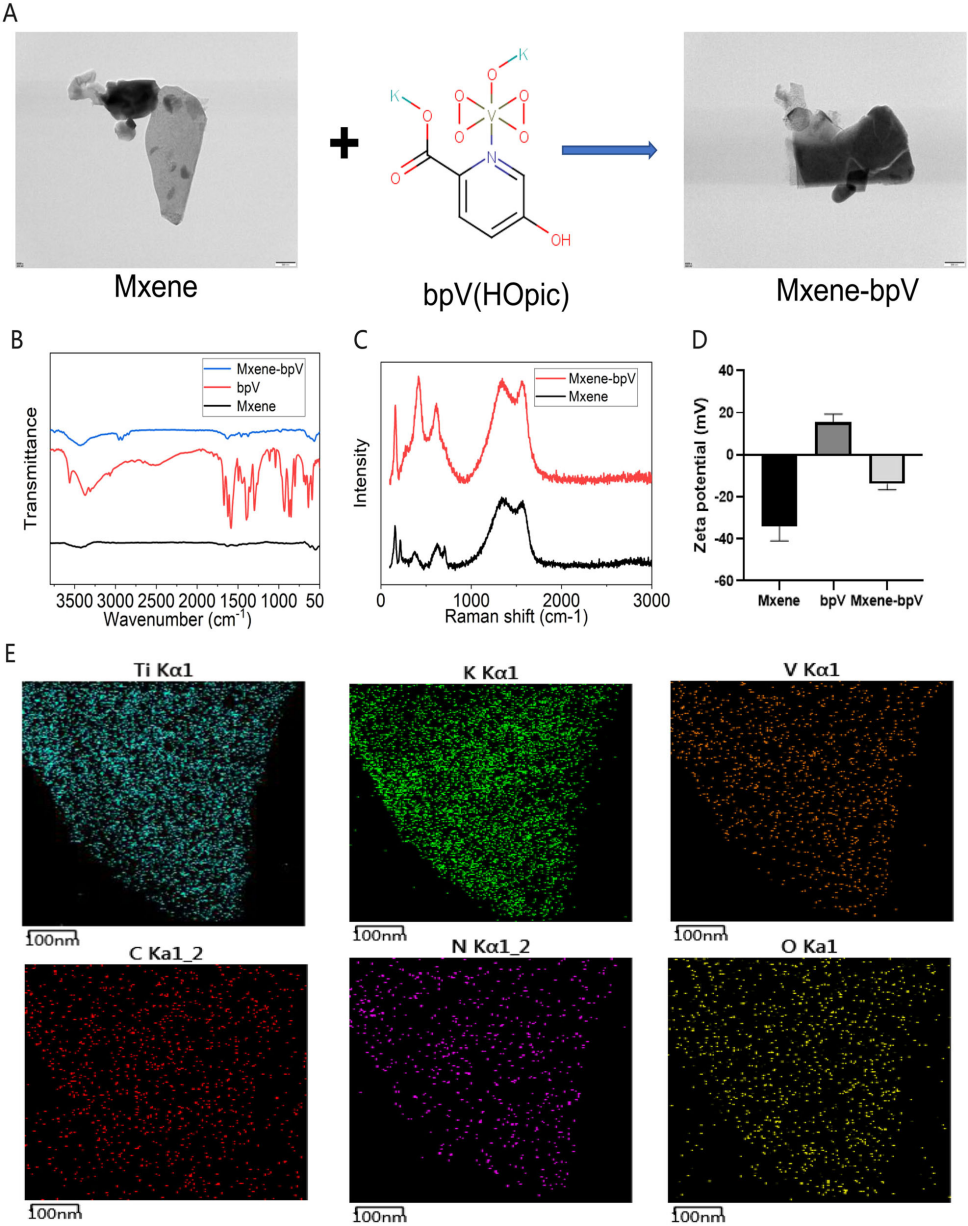
The synthesis and characterization of Mxene-bpV. **A** The structure of the prepared Mxene-bpV, as verified using transmission electron microscopy. **B** Apparent changes in Mxene-bpV at 3489 cm^–1^, as determined using infrared experiments. **C** Apparent changes in Mxene-bpV at 414 cm^–1^, as determined using XRD tests. **D** A significant reduction in the negative charge of Mxene after it was combined with bpV, as determined using zeta potential experiments. **E** EDS experiments showing the distribution of various elements in the Mxene-bpV complex

As a layered two-dimensional material, Mxene (Ti_3_C_2_T_x_) has an ultra-thin sheet structure. Here, the structure of Mxene was verified through visualization with transmission electron microscopy (Fig. 1A). The width of the Mxene sheet, as observed, was about 2–4 μm.

Mxene-bpV drug-loaded nanosheets were prepared through electrostatic adsorption between Mxene and bpV. Combining Mxene with the bpV drug significantly weakened the traditional lightness of the nanosheets during the TEM characterization process, possibly an outcome of the drug loading.

To verify the successful combination of the two, changesinMxene-bpVwereprobed at 3489 cm^–1^with the infrared experiment (Fig. 1B). The XRD test (Fig. 1C) revealedapparentchanges in Mxene-bpV at the wavelength of 414 cm^-1^, confirming the stable coupling ofMxenewithbpV.In addition, Mxene’snegative charge and bpV’s positive charge were assessed for changes using the zeta potential experiment.The negative charge of Mxene reduced significantly after the blending of Mxene with bpV(Fig. 1D), indicating that Mxenesuccessfully combined with bpV.Also, Mxeneharbors specific Ti and C elements, whilebpVis home to specific K, O and V elements. These were examined with the energy spectrum EDS experiments (Fig. 1E and Supplementary Fig. 1A, B),and the findings showed that bpV on Mxene-bpVwas very uniformly distributed on Mxene.Similarly, the EDS experimental evaluation of the distribution and content of each element on Mxene-bpV(Supplementary Fig. 1C) uncovered aneven distribution and effective loadingofbpV on Mxene.

### Mxene-bpV inhibits PTEN enzyme activity

**Fig. 2:**
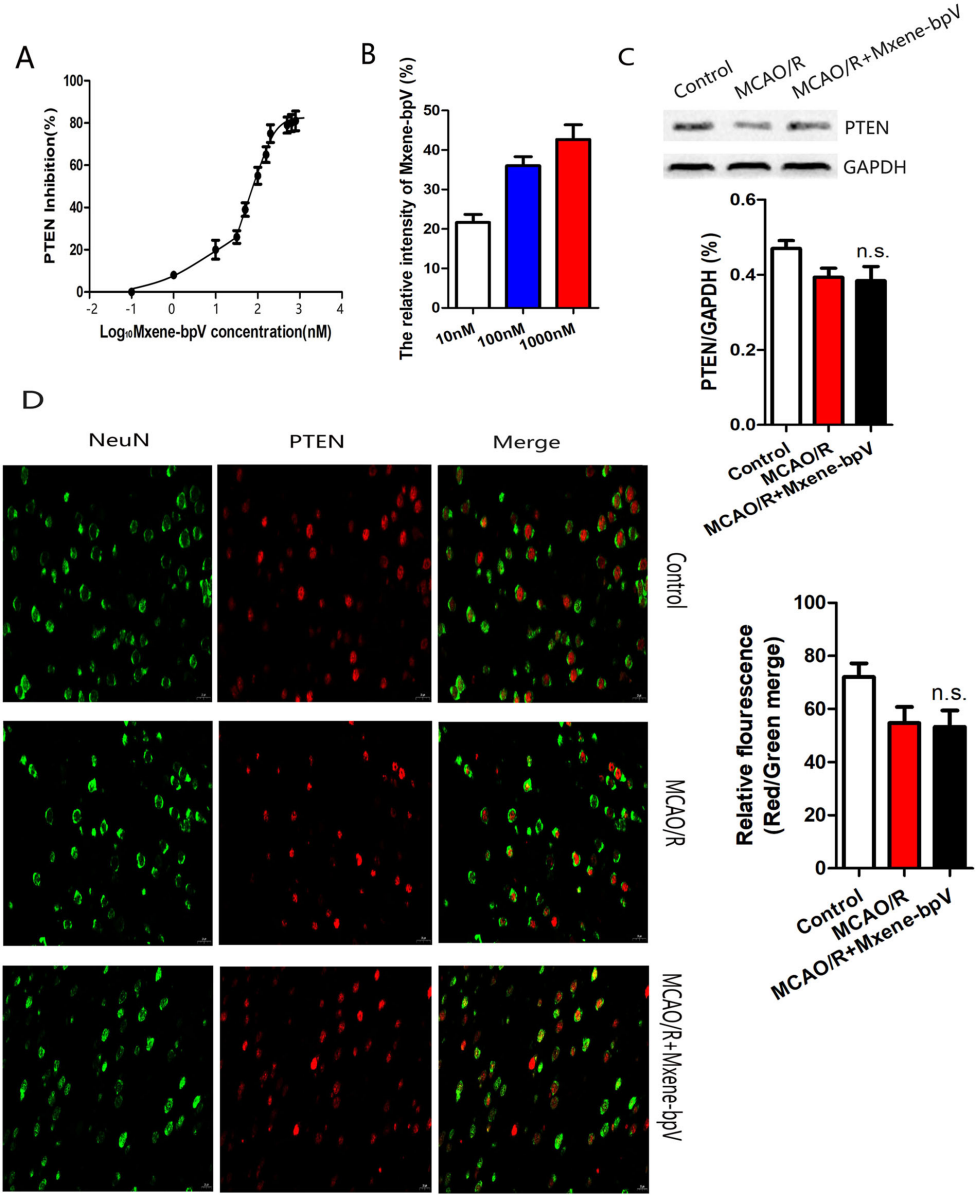
Mxene-bpV inhibits PTEN enzyme activity. **A** The percentage of PTEN activity inhibited by Mxene-bpV, as determined using the malachite green-based phosphatase assay. The Mxene-bpV of PTEN with an IC_50_ value of 31.27 ± 4.36 nM. **B** Mxene-bpV passage through the cell membrane of SH-SY5Y cells. The signal intensities of [^13^C]-Mxene-bpV and Mxene-bpV in SH-SY5Y cell extracts treated with (10, 100 and 1000 nM) Mxene-bpV, as detected using the mass spectrometry system, shows the intensity ratios of these signals relative to [^13^C]-Mxene-bpV. (*n* = 6 independentcultures). **C** The levels of the PTEN protein in mice after MCAO/R (90 min/24 h), as determined using Western blotting. (*n* = 6 in each group; n.s., no significance compared to MCAO/R). **D** Changes in PTEN expression, as determined using Double immunofluorescence staining to analyze PTEN and the neuronal marker NeuN (*n* = 6 in each group;n.s., no significance compared to MCAO/R; the one-way ANOVA test, followed by the Bonferroni post hoc test). Control: normal mice brain tissues; MCAO/R: middle cerebral artery occlusion 90 min/reperfusion of mice; MCAO/R+Mxene-bpV: mice treated intravenously with 100 nM Mxene-bpV after middle cerebral artery occlusion 90 min/reperfusion

To determine if Mxene-bpV can exert a neuroprotective effect by inhibiting PTEN enzyme activity, Mxene-bpV was probed first for its ability to inhibit PTEN activity using the malachite green-based phosphatase assay. The result was an IC_50_ value of 31.27 ± 4.36 nM (Fig. 2A), suggesting that Mxene-bpV is a potentially potent inhibitor of PTEN.

Next, Mxene-bpV’s ability to pass through the cell membrane was assessed. First, [^13^C]-Mxene-bpV- and Mxene-bpV-treated SH-SY5Y cell extracts were placed in the LC-MS system, and the signal intensities of [^13^C]-Mxene-bpV and Mxene-bpV in the cell extracts were determined. The ratio of the intensities of these signals relative to [^13^C]-Mxene-bpV (internal standard) was calculated: a high ratio denotes a high degree of Mxene-bpV passage through the cell membrane. The results are shown in Fig. 2B, where the signal intensities detected in cells treated with Mxene-bpV at 10, 100, and 1000 nM concentrations point to Mxene-bpV crossing the cell membranes.

To further explore Mxene-bpV’s ability to affect the level of PTEN protein expression in cerebral ischemia/reperfusion injury, an in vivo mouse MCAO/R injury model was set up. Western blotting registered no significant change in the expression of the PTEN protein in the intraventricular injection of Mxene-bpV mice group compared to the MCAO/R group (Fig. 2C), as did the double immunofluorescence experiment. Also, the dual immunofuorescence staining for PTEN and the neuron marker NeuN yielded no considerable change in the PTEN protein expression in the MCAO/R+Mxene-bpV group compared to the MCAO/R group (Fig. 2D). In vitro, results from the OGD/R injury model of neurons were consistent with the in vivo findings. The change in the expression of the PTEN protein in neurons in the Mxene-bpV group was not substantial relative to the OGD/R group (Supplementary Fig. 2). These results suggest that Mxene-bpV inhibited PTEN enzyme activity without affecting the level of PTEN protein expression.

### Mxene-bpV activated the Akt signaling pathway in cerebral ischemia/reperfusion injury by inhibiting PTEN

**Fig. 3:**
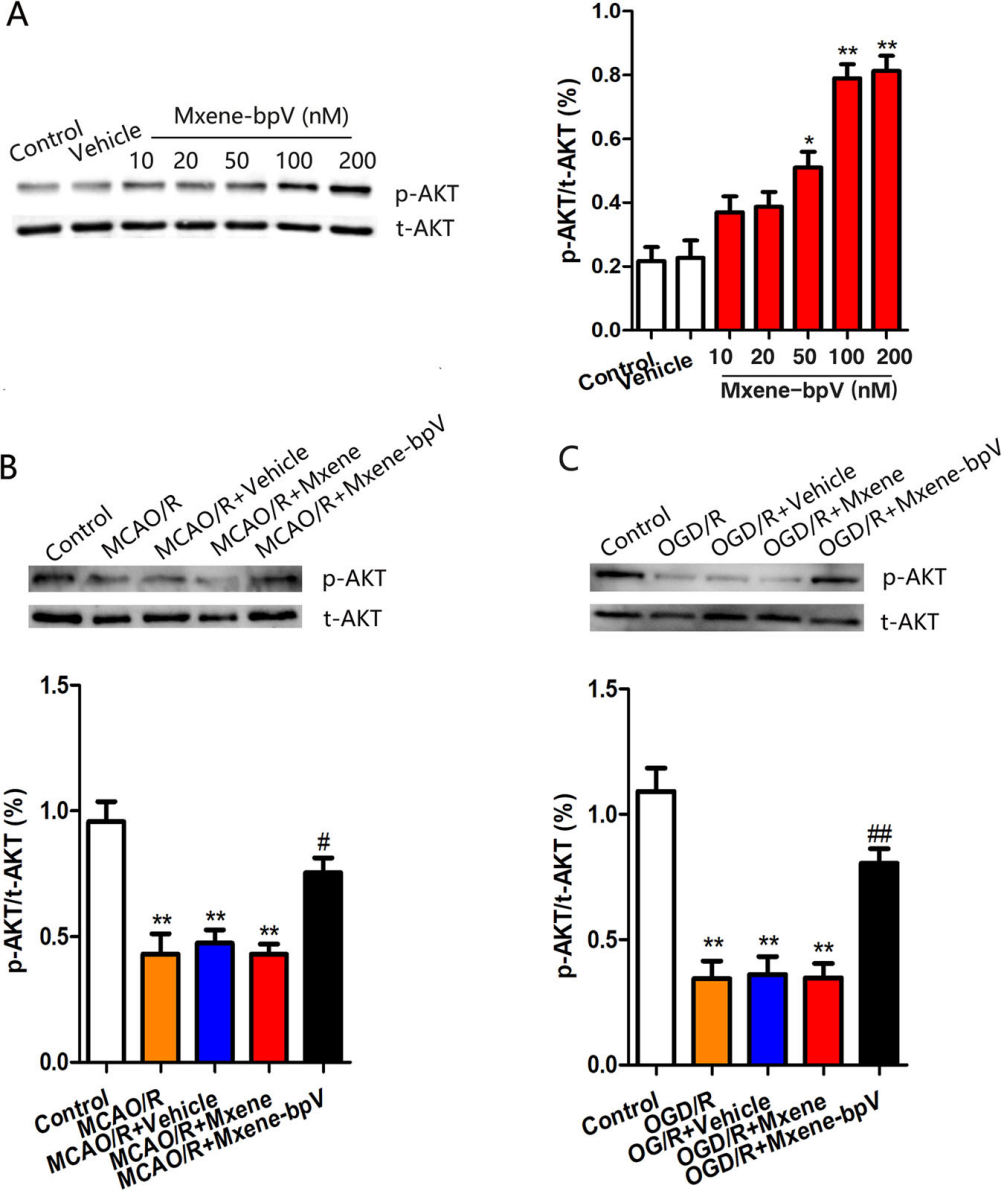
Mxene-bpV activates the Akt signaling pathway by inhibiting PTEN in cerebral ischemia/reperfusion injury. **A** Increased p-Akt levels in SH-SY5Y cells treated with Mxene-bpV (10–200 nM) (*n* = 6) independent cultures; *p < 0.05 or **p < 0.01 compared to Vehicle; the one-way ANOVA test, followed by the Bonferroni post hoc test. Control: normal SH-SY5Y cells; Vehicle: SH-SY5Y cells treated with the same volume of saline but with no Mxene-bpV; Mxene-bpV: SH-SY5Y cells treated with different concentrations of Mxene-bpV, from 10 to 200 nM. **B** The levels of the p-AKT protein in mice after MCAO/R in different groups, as determined using the Western blot assay (*n* = 6 for each group; **P < 0.01 compared to Control; ^#^*P* < 0.05 compared to MCAO/R; the two-way ANOVA test, followed by the Bonferroni post hoc test). **C** The levels of the p-AKT protein in primary cultured micecortical neurons after OGD/R in different groups, as determined using the Western blot assay (*n* = 6 for each group; **P < 0.01 compared to Control; ^##^*P* < 0.01 compared to OGD/R; the two-way ANOVA test, followed by the Bonferroni post hoc test). Control: normal mice cortical neurons; OGD/R: neurons transferred to a deoxygenated, glucose-free extracellular solution for 90 min/restore oxygen and glucose

As PTEN regulates Akt activation negatively [21], activating Akt could be a protective play in ischemia-reperfusion injury [22]. To explore the mechanism of Mxene-bpV in cerebral ischemia/reperfusion injury further, Mxene-bpV was scrutinized for its ability to exert an effect on Akt activation in vivo and in vitro. Akt activity was quantified via the Western blotting of Akt phosphorylation on Ser^473^ (p-Akt) [14]. Firstly, the most significant increase in p-Akt levels in SH-SY5Y cells treated with 100 nM Mxene-bpV was noted using Western blotting (Fig. 3A).

For the in vivo study, MCAO/R mice received Mxene-bpV injections through the lateral ventricle, while the control group was given the same volume of saline through the lateral ventricle. Compared to the control group, the levels of p-Akt in the group administered Mxene-bpV increased drastically, but no significant change was observed in the group given the same volume of Mxene (Fig. 3B). Comparable results were obtained in primary mouse cortical neurons. In the established OGD/R injury model in mice cortical neurons, p-Akt levels were extensively higher in the group treated with Mxene-bpV than in the group treated with the same volume of the saline Vehicle (OGD/R+Vehicle) (Fig. 3C). These results indicate that Mxene-bpV activated Akt in cerebral I/R injury by inhibiting PTEN.

### Mxene-bpV promotes M2 microglia polarization and reduces inflammatory responses in ischemia/reperfusion injury

**Fig. 4:**
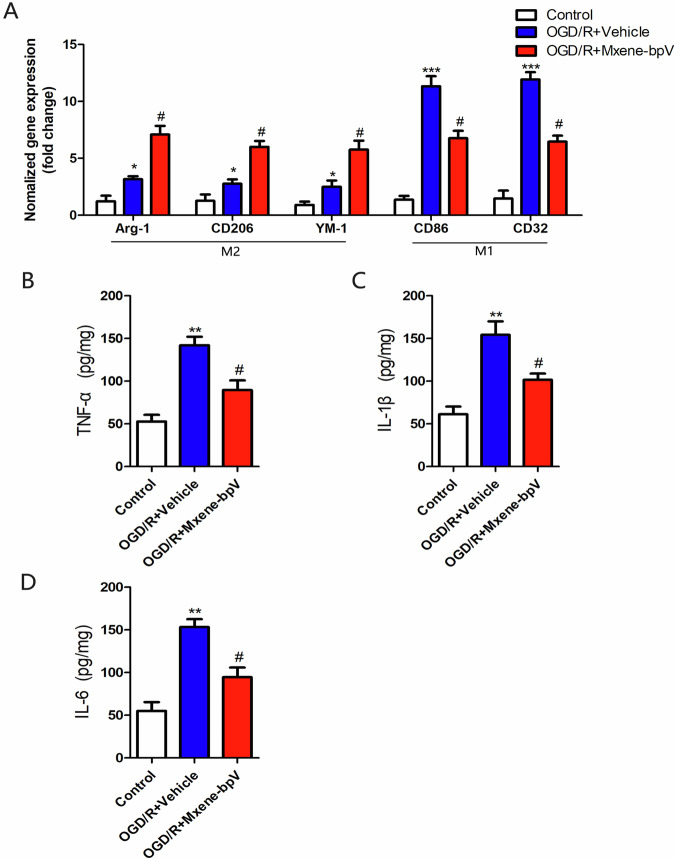
Mxene-bpV promotes M2 microglia polarization. **A** The qRT-PCR analysis of the primary cultures of microglia for markers of M1 and M2 microglia after OGD/R. **B**–**D** Levels of TNF-α, IL-1β and IL-6 in microglia after OGD/R, as determined using ELISA (*n* = 6 in each group; *p < 0.05, **p < 0.01, or ***p < 0.001 compared to Control; ^#^*p* < 0.05 compared to OGD/R + Vehicle). Statistical differences were determined using the two-way ANOVA test, followed by the Bonferroni post hoc test

Mxene-bpV was further examined in vitro in order to explore its effect on anti-inflammatory marker-M2 microglia polarization and inflammatory response. The mRNA levels of M1 and M2 microglia markers in mice cortical neurons were measured after OGD/R injury using the qRT-PCR analysis. Compared to the OGD/R+Vehicle group, the levels of M2 microglia markers Arg-1, CD206 and YM1 increased in the presence of Mxene-bpV [23, 24], while the levels of M1 microglia markers CD86 and CD32 reduced after Mxene-bpV treatment, but not statistically significantly (Fig. 4A). These results show that Mxene-bpV promoted M2 microglia polarization.

Additionally, comparison with the Mxene treatment group and the bpV treatment group revealed that Mxene-bpV treatment had the most considerable effect on the increase in the levels of YM1, CD206 and Arg-1 (Supplementary Fig. 3A–C).

To determine if Mxene-bpV can ultimately regulate the release of inflammatory cytokines under the experimental conditions in this investigation, TNF-α, IL-1β and IL-6 were used as indicators to monitor the levels of inflammatory factors. As shown in Fig. 4B–D, treatment with Mxene-bpV clearly inhibited the elevation of TNF-α, IL-1β, and IL-6. Taken together, these results suggest that Mxene-bpV promotes M2 microglia polarization and reduces inflammatory responses.

### Mxene-bpV promotes M2 microglia polarization by inhibiting PTEN to reduce inflammation

Studies have shown previously that PTEN plays an anti-inflammatory role by activating the anti-inflammatory pathway of M2 macrophages [25]. Here, various experiments were performed to explore Mxene-bpV’s ability to regulate microglia via PTEN in order to affect neuronal survival.

**Fig. 5:**
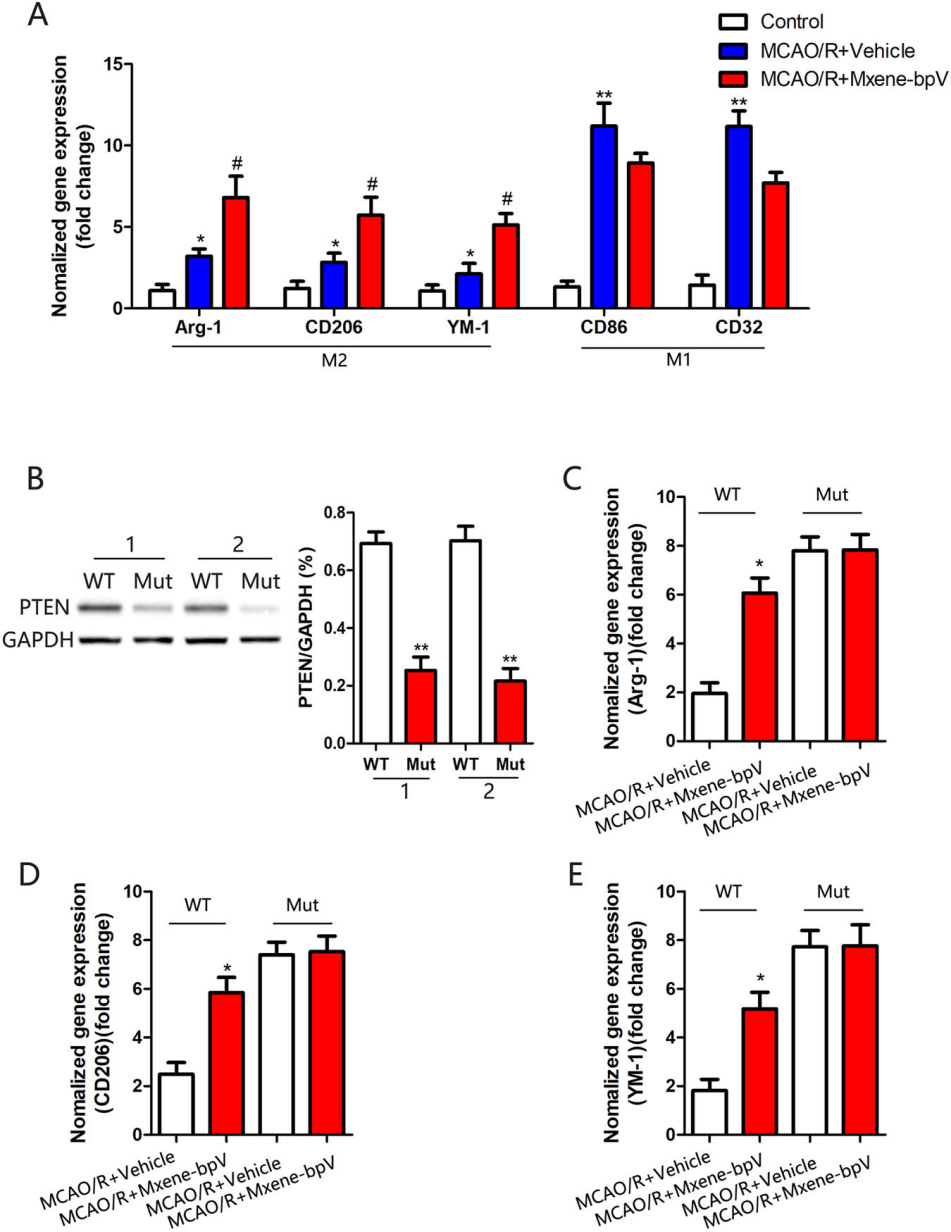
Mxene-bpV promotes M2 microglia polarization by inhibiting PTEN, thereby reducing inflammatory responses. **A** The qRT-PCR analysis of M1and M2 microglial marker levels in mice after the MCAO/R (90 min/24 h) injury. **B** Significantly reduced PTEN protein levels in PTEN mutant mice compared to wild-type mice, as determined using Western blotting. **C** Arg-1 levels in each group, as determined using the qRT-PCR analysis. **D** The qRT-PCR analysis of CD206 levels in each group. **E** YM-1 levels in each group, as determined using the qRT-PCR analysis (*n* = 6 in each group;*p < 0.05 or **p < 0.01compared to Control, MCAO/R+Vehicle, or WT; ^#^*p* < 0.05 compared to MCAO/R + Vehicle; the two-way ANOVA test, followed by the Bonferroni post hoc test). WT: PTEN-wild-type mice. Mut: PTEN-mutant mice

Through qRT-PCR analysis, the mRNA levels of M2 and M1 microglia markers were measured in the brain tissues of the injured side cortexes of the MCAO/R injury mice. Compared to the MCAO/R+Vehicle group, the levels of M2 microglia-specific markers, Arg-1, CD206 and YM1, increased after treatment with Mxene-bpV, and the levels of M1 microglia-specific markers, CD86 and CD32, reduced after Mxene-bpV treatment, but not statistically notable (Fig. 5A). These results show that Mxene-bpV promoted M2 microglia polarization.

To prove that Mxene-bpV promotes M2 microglia polarization by inhibiting PTEN further, recombinant PTEN transgenic mice were established using tamoxifen and then put through experimentation. Such mice usually coexist with different proportions of PTEN wild-type and PTEN knockout cells, with PTEN not removed from all cells; therefore, these mice are referred to as “PTEN mutant” mice. Here, PTEN expression reduced significantly in PTEN-mutant(Mut) mice (Fig. 5B). Mxene-bpV treatment caused an increase in the levels of YM-1, CD206 and Arg-1 in MCAO/R-injured wild-type mice but not in MCAO/R-injured PTEN-mutant mice (Fig. 5C, D). These results suggest that Mxene-bpV promotes M2 microglia polarization by inhibiting PTEN.

Next, Mxene-bpV’s ability to reduce inflammation was probed. The protein levels of proinflammatory cytokines, including TNF-α, IL-1β and IL-6, were measured. Treatment with Mxene-bpV pointedly inhibited increases in TNF-α, IL-1β and IL-6 (Supplementary Fig. 4A–C).

Taken together, these results imply that Mxene-bpV promotes M2 microglia polarization by inhibiting PTEN to reduce inflammation.

### Mxene-bpV plays a neuroprotective role in cerebral ischemia-reperfusion injury

**Fig. 6:**
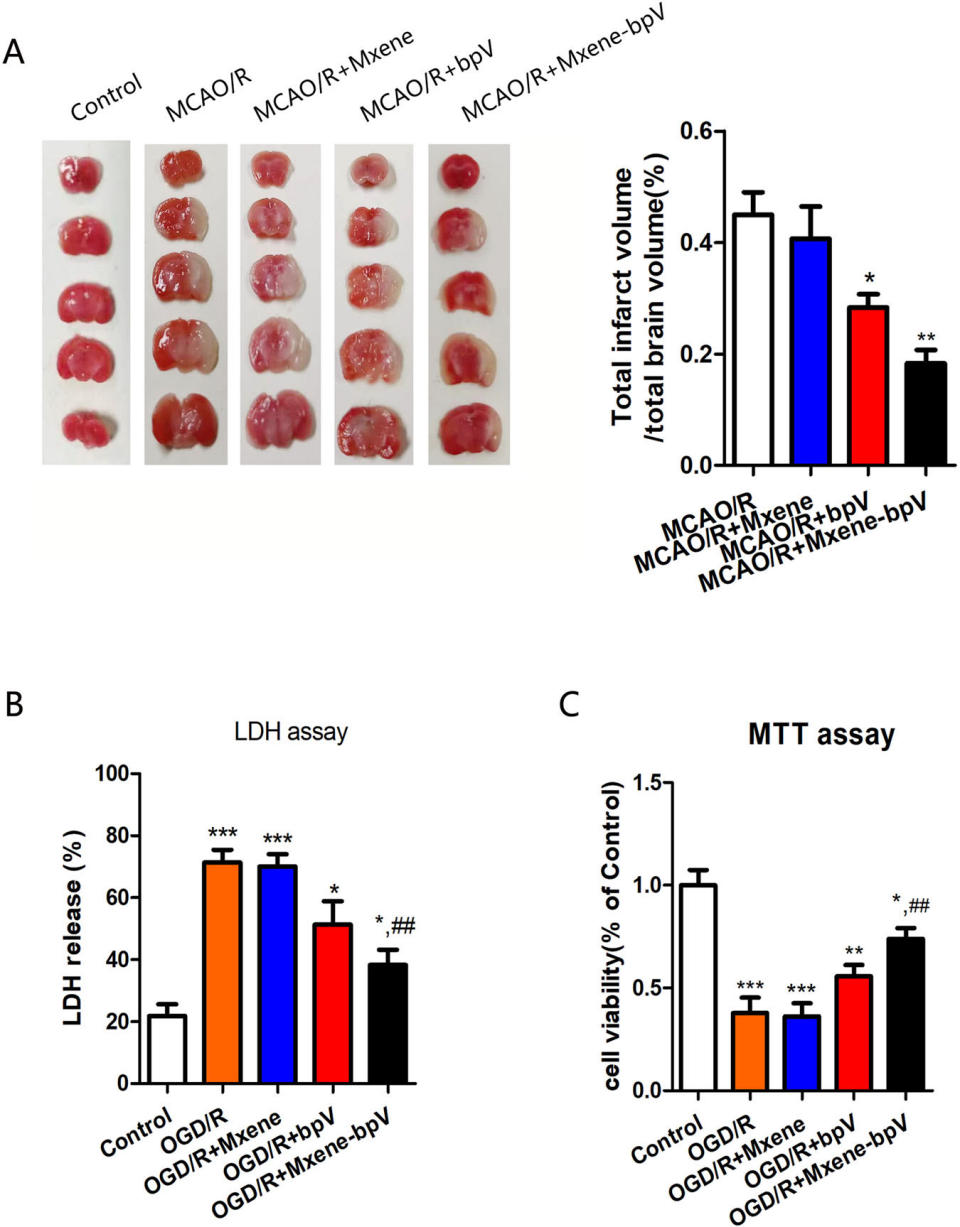
Mxene-bpV plays a neuroprotective role in cerebral ischemia-reperfusion injury. **A** Infarct volumes in the rat brains in each group after MCAO/R (90 min/24 h), as determined using TTC staining. **B** Mxene-bpV-instigated reduced release of the OGD-induced cortical neuronal cytotoxic substance, LDH, as determined using the LDH assay. **C** The cortical neuronal survival rate in each group, as determined using the MTT assay (*n* = 6 in each group; *p < 0.05, **p < 0.01 or ***p < 0.001 compared to MCAO/R or Control; ^#^*p* < 0.05 compared to OGD/R; the two-way ANOVA test, followed by the Bonferroni post hoc test)

Finally, to verify Mxene-bpV’s ability to play a neuroprotective role in I/R injury, the MCAO/R injury model was validated. 2,3,5-triphenyltetrazolium chloride (TTC) staining data showed that Mxene-bpV treatment most notably decreased infarct volumes 24 h after MCAO (Fig. 6A). Comparable results were obtained in cultured neurons. Mxene-bpV treatment noticeably boosted neuronal viability and lowered LDH release (Fig. 6B, C).

**Fig. 7:**
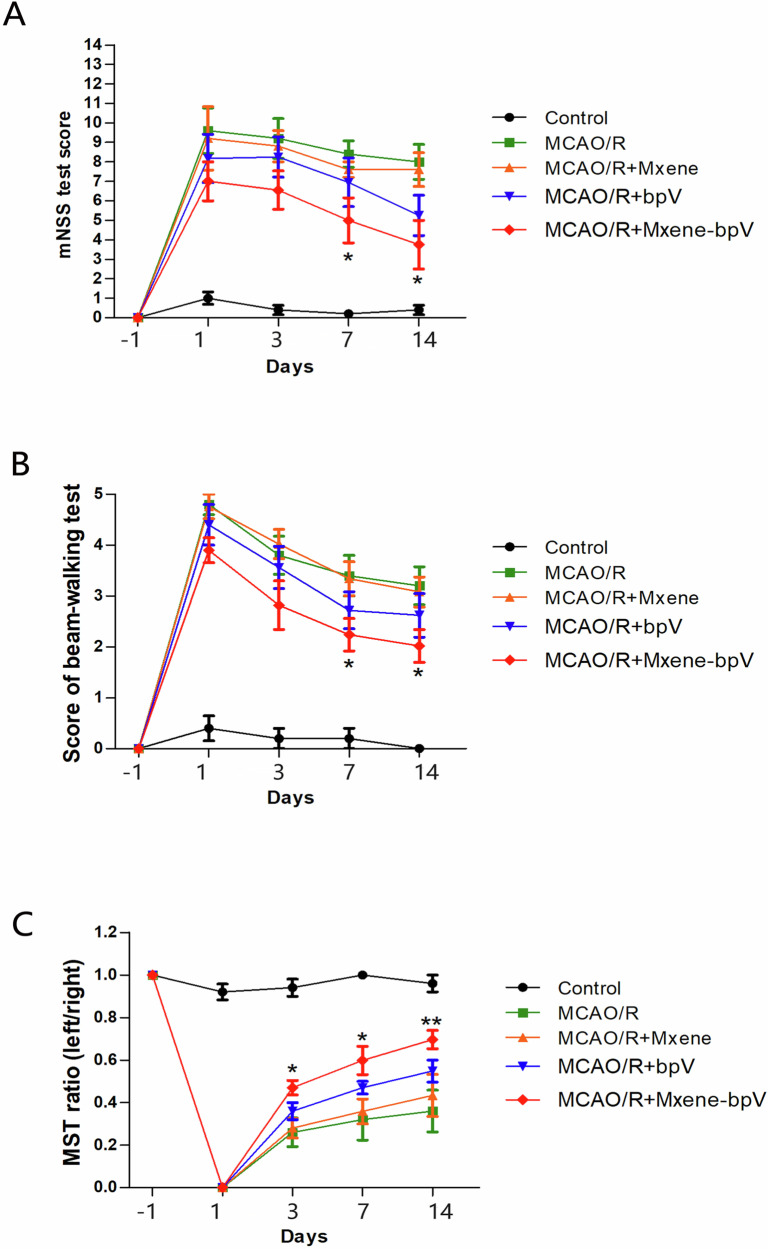
Mxene-bpV promotes the recovery of brain function after cerebral ischemia-reperfusion injury in mice. **A** Mxene-bpV-treated mice’s low scores on days 7 and 14 post-MCAO/R, as determined using the mNSS test. **B** Mxene-bpV-treated mice’s low scores on days 7 and 14 after MCAO/R, as determined using the Beam walking test. **C** Mxene-bpV-treated mice’s high ratios on days 3, 7 and 14 after MCAO/R, as determined using the MST test (*n* = 6 for each group; *p < 0.05 or **p < 0.01 compared to MCAO/R; the two-way ANOVA test, followed by the Bonferroni post hoc test)

Next, a series of behavioral experiments were conducted to validate the neuroprotective effect of Mxene-bpV on cerebral I/R-injured animals. Compared to mice in the MCAO/R group, mice treated with Mxene-bpV scored lower in the mNSS test and Beam walking test and higher in the MST test on days 3, 7 and 14 (Fig. 7A–C). Conclusively, Mxene-bpV promoted the recovery of brain function and played a neuroprotective role in mice with cerebral ischemia/reperfusion injury.

## Discussion

Among currently tested biomaterials, Mxenes have been successfully applied in biomedical fields, including in nanomedicine, biosensors, antimicrobial therapy, self-cleaning, bioimaging, and therapeutic diagnostics, thanks to its excellent electrical conductivity and hydrophilicity [26–28]. Increasingly more studies have proven that the two-dimensional structure of Mxenes is rich in surface anchoring sites, giving them enormous potential as drug carriers [29]. Moreover, recent research has found Mxenes to be biocompatible and to be non-cytotoxic in neural stem cells [30]. Mxenes have also been recorded in action in in vivo nerves, and free cortical neurons cultured on Mxenes can adhere normally, grow neurites and form neural networks [31].

**Fig. 8:**
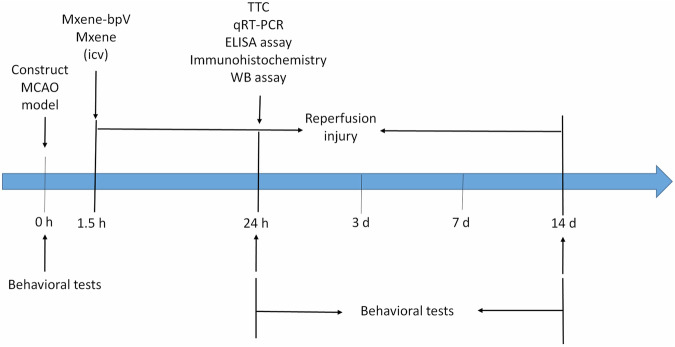
Flow chart of animal experiments

In this study, synthesized Mxene-loaded bpV (HOpic) was used to treat cerebral I/R injury for the first time. Per the results obtained, the compound was stable and easily crossed the cell membrane Fig. 8. Treating cerebral I/R injury with Mxene-bpV revealed that Mxene-bpV treatment significantly increased the survival rate of injured neurons, reduced infarct sizes, and promoted the recovery of brain function in mice with I/R injury compared to bpV (HOpic) treatment alone. This could be in association with the excellent electrical conductivity, hydrophilicity and biocompatibility of Mxene. Mxene-bpV’s role was also probed in primary cultured mice cortical neurons, expanding the understanding of the application of Mxene in the field of nervous system diseases.

PTEN is a tumor suppressor that plays a crucial role in inhibiting tumor growth [32, 33]. Previous research has shown that inhibiting the lipid phosphatase activity of PTEN activates Akt, and inhibiting PTEN protein phosphatase activity restrains extra synaptic GluN2B-containing NMDA receptors, thereby reducing the mortality of ischemic neurons [7]. In this study, Mxene-bpV, a new nanocomposite formed by loading Mxene(Ti_3_C_2_T_x_) with bpV(HOpic), was assessed in the treatment of cerebral I/R injury for the first time. Bisperoxovanadium (bpV), a derivative of vanadate, is a putative inhibitor of PTEN. Mxene-bpV, through PTEN inhibition and Akt activation, was more effective than bpV(HOpic) alone in reducing cerebral infarct sizes and promoting brain function recovery after MCAO/R injury in mice. Comparable results were obtained in the in vitro experiments. The efficiency of Mxene-bpV treatment in curbing the mortality of mouse cortical neurons after OGD/R was superior to that of bpV treatment alone, an outcome consistent with previous findings that PTEN inhibition has a neuroprotective effect in cerebral ischemia/reperfusion [34].

Usually, an inflammatory response increases brain damage after cerebral ischemia-reperfusion injury: a proinflammatory response leads to tissue damage, blood-brain barrier disruption, secondary edema, and brain cell death [35]. This study established the first evidence of Mxene-bpV’sneuroprotective effects against cerebral ischemia-reperfusion injury via a reduction in the levels of pro-inflammatory cytokines, including IL-1β, IL-6 and TNF-α. For the first time also, Mxene-bpV was revealed to be neuroprotective against I/R injury by mediating anti-inflammatory responses.

Recent investigations have shown that the inhibition of PTEN activates anti-inflammatory pathways in M2 peripheral macrophages [25]. In this probe, qRT-PCR was used to assess the mRNA levels of genes associated with the activation of M1 or M2, with anti-inflammatory mediators Arg-1, CD206, and YM-1, which can prevent inflammation and protect nerves, selected as gene markers for the activation of M2 microglia [23, 36], and CD86 and CD32, which are pro-inflammatory, selected as gene markers for the activation of M1 microglia [23, 24].The findings of the current research matched those revelations: the inhibition of PTEN promoted M2 microglial polarization. Because the anti-inflammatory mediators, Arg-1, CD206 and YM-1, expressed by M2 microglia can prevent inflammation and provide neuroprotection [36], the three mediators were selected as markers and used to evaluate Mxene-bpV’s ability to exert its neuroprotective effect through the promotion of M2 microglial polarization. The results obtained suggest that Mxene-bpV enhanced the anti-inflammatory impact of M2 microglia, promoting survival in I/R injury.

To further ensure that Mxene-bpV promotes M2-type microglial polarization by inhibiting PTEN, PTEN transgenic mice were put in place and subjected to experiments. Accordingly, the levels of Arg-1, CD206 and YM-1 increased in MCAO/R-injured wild-type mice after treatment with Mxene-bpV, but not in MCAO/R-injured PTEN-mutant mice (Mut) (Fig. 5C, D), suggesting that Mxene-bpV promotes M2 microglia polarization by inhibiting PTEN.

This study highlights a new concept for the application of Mxene in the development of drug carriers for the treatment of nervous system diseases. It also advances compelling evidence for the application of the new nanosynthetic Mxene-bpV in the treatment of cerebral ischemia-reperfusion injury, as well as a new direction for the treatment of ischemic stroke.

## Supplementary information

Supplementary Figure 1

Supplementary Figure 2

Supplementary Figure 3

Supplementary Figure 4

Supplementary Figure Legend
