# Supplementary figures and images for: Mxene-bpV plays a neuroprotective role in cerebral ischemia-reperfusion injury by activating the Akt and promoting the M2 microglial polarization signaling pathways
Source: J Mater Sci Mater Med. 2024 Jul 29;35(1):42. doi: 10.1007/s10856-024-06811-0 (PMC11286715; doi:10.1007/s10856-024-06811-0)

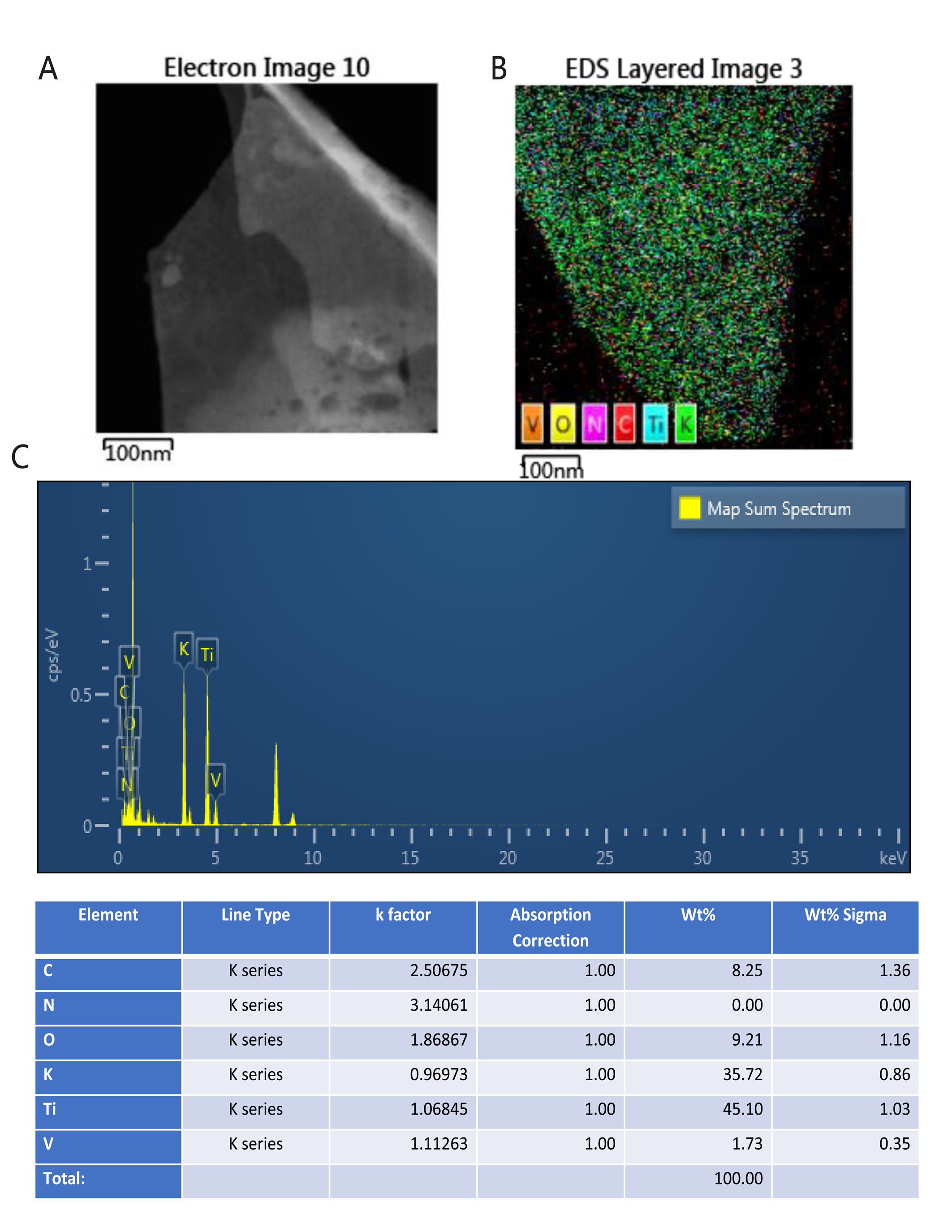

Supplement: Supplementary file 1 — Supplementary Figure 1 [file 10856_2024_6811_MOESM1_ESM.jpg]

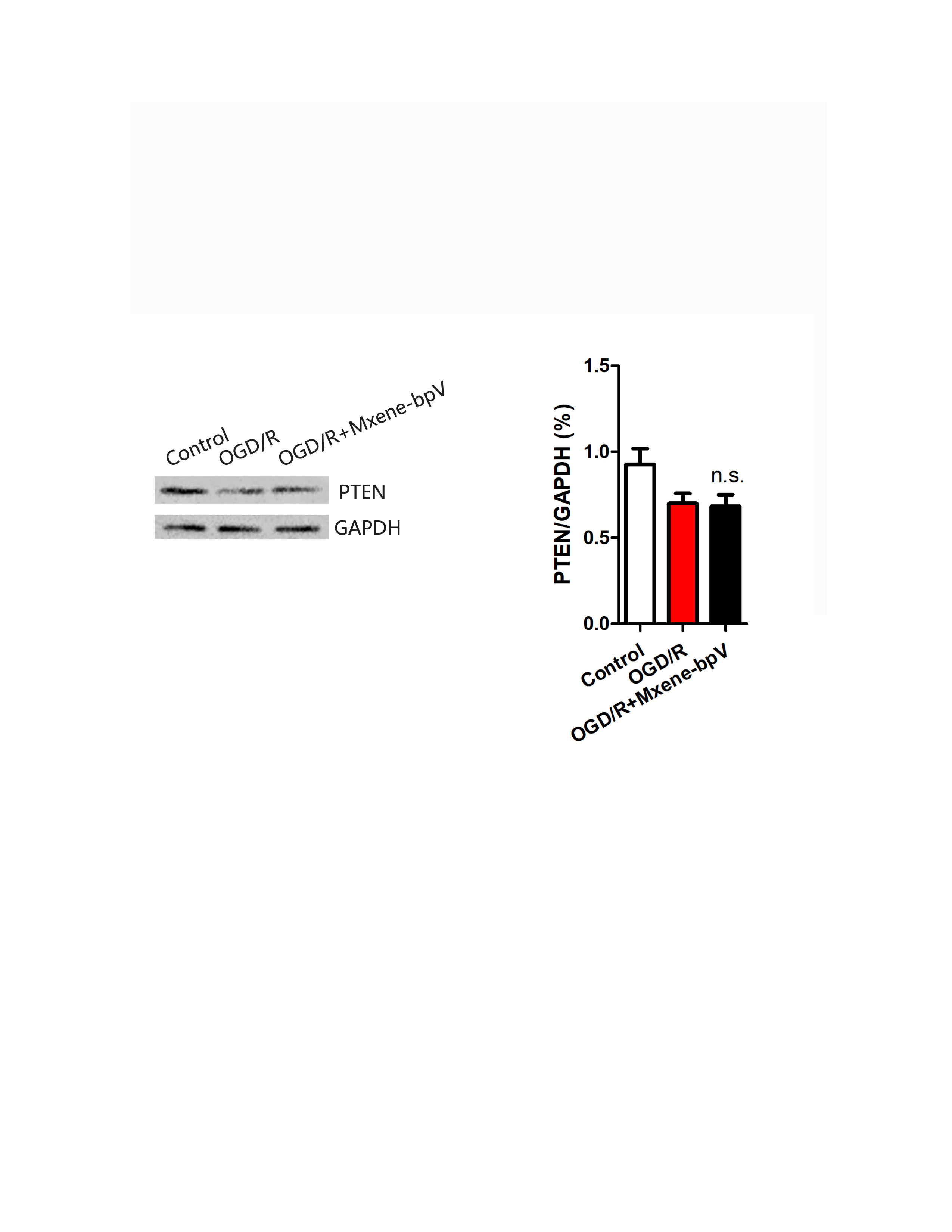

Supplement: Supplementary file 2 — Supplementary Figure 2 [file 10856_2024_6811_MOESM2_ESM.jpg]

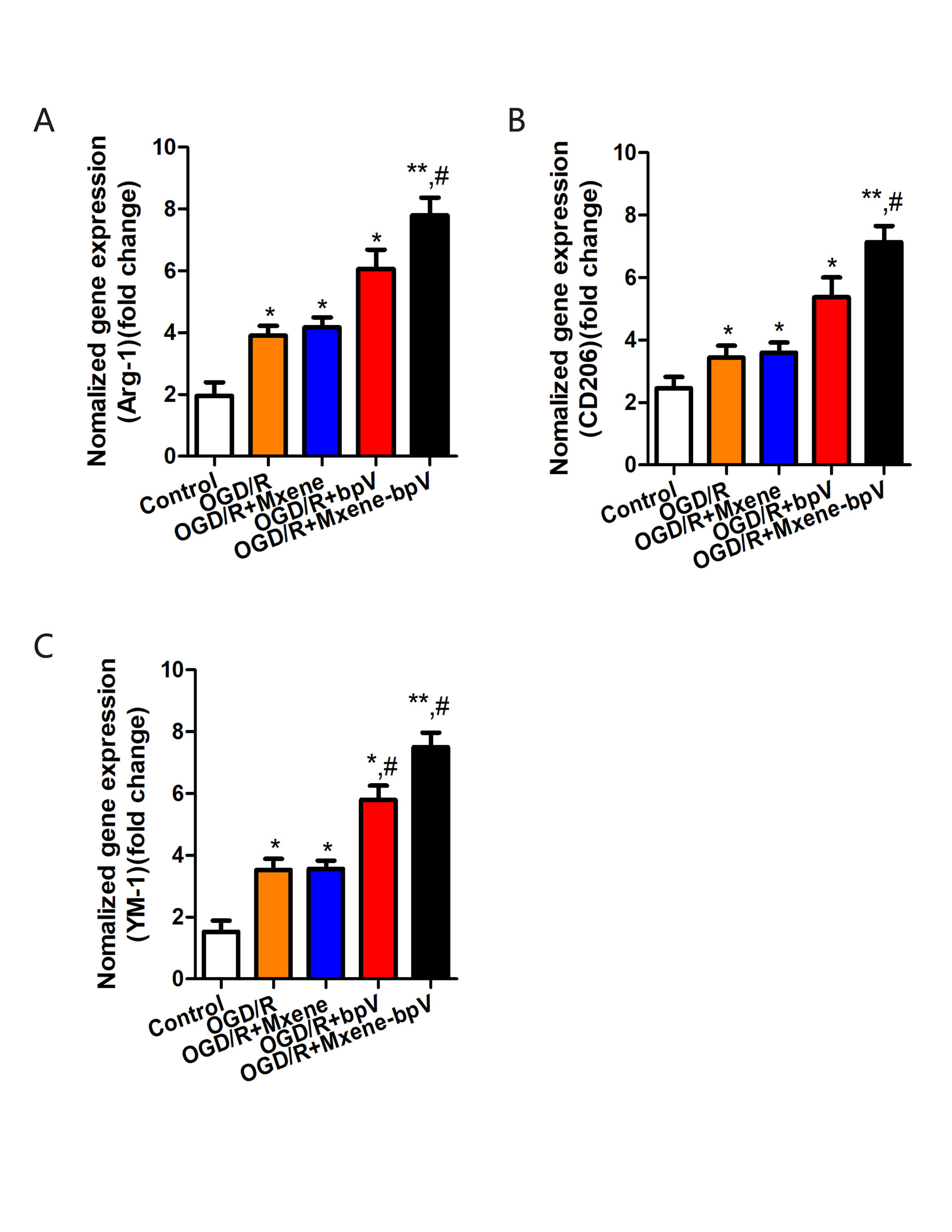

Supplement: Supplementary file 3 — Supplementary Figure 3 [file 10856_2024_6811_MOESM3_ESM.jpg]

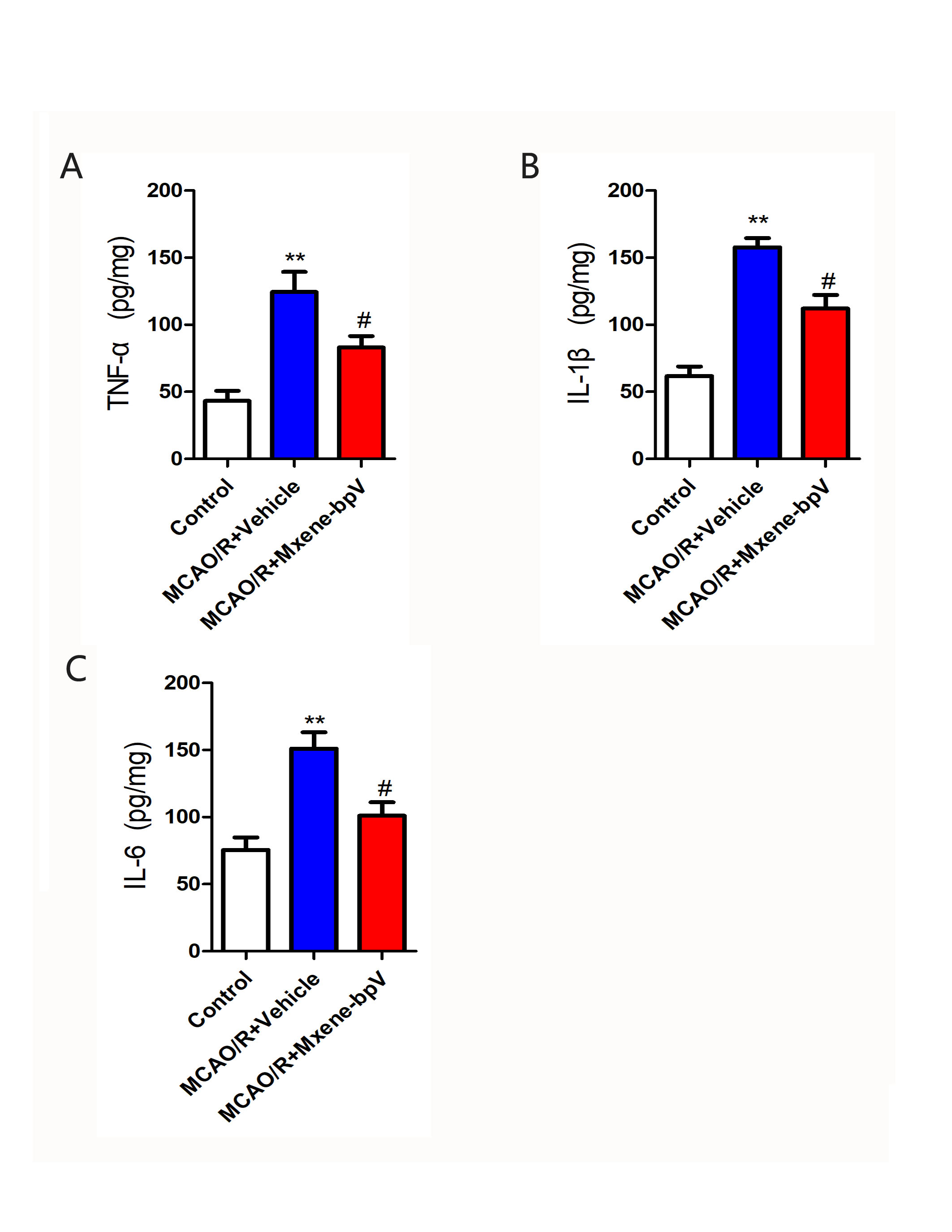

Supplement: Supplementary file 4 — Supplementary Figure 4 [file 10856_2024_6811_MOESM4_ESM.jpg]
