# Supplementary material for: Mxene-bpV plays a neuroprotective role in cerebral ischemia-reperfusion injury by activating the Akt and promoting the M2 microglial polarization signaling pathways
Source: J Mater Sci Mater Med. 2024 Jul 29;35(1):42. doi: 10.1007/s10856-024-06811-0 (PMC11286715; doi:10.1007/s10856-024-06811-0)
Supplement: Supplementary file 5 — Supplementary Figure Legend [file 10856_2024_6811_MOESM5_ESM.docx]

Supplementary Fig. 1. (A-B) A very uniform distribution on Mxene, as determined with EDS experiments on bpV on Mxene-bpV. (C) The distribution and content of each element on Mxene-bpV, as determined using EDS experiments.

Supplementary Fig. 2. Mxene-bpV did not affect the expression of the PTEN protein. The levels of the PTEN protein in primary cultured mouse cortical neurons after OGD/R, as determined using the Western blot assay (n = 6 in each group; n.s., no significance compared to OGD/R; the two-wayANOVA test, followed by the Bonferroni *post hoc* test).

Supplementary Fig. 3. (A) A qRT-PCR analysis showing Arg-1 levels in each group. (B) A qRT-PCR analysis showing CD206 levels in each group. (C) A qRT-PCR analysis showing YM-1 levels in each group (n = 6 in each group; *p <0.05 or **p <0.01compared to Control; #p < 0.05 compared to OGD/R; the two-way ANOVA test, followed by the Bonferroni post hoc test).

Supplementary Fig. 4. (A) The levels of pro-inflammatory cytokine TNF-α in mice 24 h after MCAO/R, as determined using the ELISA assay. (B) The levels of IL-1βin mice 24 h after MCAO/R, as determined using the ELISA assay. (C) The levels of IL-6in mice 24 h after MCAO/R, as determined using the ELISA assay (n = 6 in each group; **p <0.01compared to Control; ^#^p < 0.05 compared to MCAO/R + Vehicle). Statistical differences were calculated using the two-way ANOVA test, followed by the Bonferroni *post hoc* test.
